# Editorial: New Approaches Against Drug-Resistant *M. tuberculosis*

**DOI:** 10.3389/fmicb.2021.681420

**Published:** 2021-04-15

**Authors:** Yossef Av-Gay, Giorgia Mori, Maria Rosalia Pasca

**Affiliations:** ^1^Departments of Medicine and Microbiology & Immunology, The University of British Columbia, Vancouver, BC, Canada; ^2^The University of Queensland Diamantina Institute, The University of Queensland, Woolloongabba, QLD, Australia; ^3^Department of Biology and Biotechnology “Lazzaro Spallanzani”, University of Pavia, Pavia, Italy

**Keywords:** tuberculosis, host directed therapy, drug discovery, antimicrobial resistance, drug targets, anti-tubercular drugs

Tuberculosis (TB) is the most devastating infectious disease worldwide, killing 1.4 million people each year (World Health Organization [WHO], 2020). Treatment regimens are lengthy and cause considerable adverse effects, leading to poor patient compliance, as well as high costs and economic burden worldwide. Moreover, resistance to newly approved antimicrobials for TB develops quickly (Bloemberg et al., 2015; Zimenkov et al., 2017). This, together with the widespread occurrence of *Mycobacterium tuberculosis* (*Mtb*) drug-resistant strains, necessitates discovery of novel and innovative drugs that will shorten the duration of TB treatments and/or be less toxic to the human host.

Our Research Topic entitled “New approaches against drug-resistant *Mtb*” called for original research papers and reviews describing novel approaches for drug discovery, target identification and mechanisms of drug resistance. The response we have received was exciting resulting in 13 articles with more than 30,000 views.

Our collection includes an exciting review describing drug development in our era where modern -omic technologies, such as DNA and RNA sequencing, proteomics, and genetic manipulation of organisms, facilitate the drug discovery process for TB treatment. These methods enable us to better understand mechanisms of action of antibiotics and allow the evaluation of new drug candidates using mathematical modeling and modern computational analysis for the drug discovery process.

*Mtb*, the causative agent of TB, infects, survives, and replicates intracellularly within alveolar macrophages. *Mtb* utilizes numerous strategies to avoid host defense mechanisms and preys on the host cell response to establish infection (Hmama et al., 2015). A novel drug discovery approach termed Host Directed Therapy (HDT), was proposed recently to overcome drug resistance (Zumla et al., 2016; Parish, 2020). Since HDTs do not target *Mtb* directly but rather assist the host in fighting infection, they would have reduced chances of generating resistance.

A series of publications within our Collection explore this new and exciting approach to TB drug development.

Shapira et al. screened a library of human kinase inhibitors and identified several compounds that are active in an intracellular model of TB infection. A specific checkpoint inhibitor showed promising activity and CHK2 inhibition by RNAi phenocopied the intracellular inhibitory effect of the drug.

Smyth et al. showed that Protein Kinase R (PKR) is a human host cell sensor that function in the cellular response to mycobacterial infection. They demonstrated that the over-expression of PKR enhanced anti-mycobacterial activity of macrophages and it is mediated by selective autophagy and by the inhibition of autophagolysosome maturation which limit *Mtb* replication, suggesting that PKR can be considered for HDT development.

Screening of compound libraries is a valuable tool in drug discovery; Pollo et al. tested a library of 52 natural and synthetic compounds for activity against *Mtb* growth and identified the natural products isobavachalcone and isoneorautenol, and a synthetic chromene as active at low micromolar concentrations.

Noschka et al., screened a library of antimicrobial peptides generated from hemo-filtrate identifying Angiogenin as the only active compound against *Mtb* growth. They confirmed its activity using the synthetic Angiogenin. They derived the small peptide fragment Angie1, which is active against intracellular and extracellular *Mtb* growth, as well as they tested that it is not toxic for zebrafish embryos.

Khara et al. assessed the anti-mycobacterial potency of three novel synthetic anti-microbial peptoides against *Mtb* growth and showed that BM2 peptoid kills mycobacteria both *in vitro* and intracellularly. More importantly, it significantly reduced bacterial load in the lungs of infected mice.

The effect of novel and alternative compounds was also described in our collection; selenium nanoparticles (by Estevez et al.) and epidioxy-sterol analogs (Baena et al. In particular, Estevez et al. demonstrated that the selenium nanoparticles are active against both *Mtb* and *Mycobacterium smegmatis* growth by targeting their cell envelope. This finding opens new perspectives because this type of nanoparticles is characterized by a low toxicity.

Baena et al. tested the activity of 15 epidioxy-sterol analogs against *Mtb* both *in vitro* and *ex vivo*. T5 epidioxy-sterol-ANB was effective against *Mtb in vitro* only inside macrophages. Furthermore, it was showed that it is active also in a *Mtb* infected murine model. By transcriptomics analysis of *Mtb* infected macrophages after T5 epidioxy-sterol-ANB treatment, a significant down-regulation of enzymes involved in the cholesterol and folic acid pathways was discovered.

Study of the mechanism of resistance and mode of action of drugs used in TB treatment as well as identification of novel resistance mutations are extremely important for our understanding of this topic and for the development of novel approaches to fight these emerging *Mtb* drug-resistant strains.

Isoniazid (INH) and rifampicin (RIF) are two first-line drugs which are extensively used in TB therapy (Lange et al., 2019). Several SNPs conferring drug resistance have already known for both compounds, but it is mandatory to continue to search for new ones.

Hsu et al. identified two novel mutations (W341R and L398P) in the known drug activator KatG conferring INH resistance. Interestingly, they showed that these two polymorphisms are responsible for INH resistance by complementation and by the construction of two mutants harboring the same mutations.

Lai et al. demonstrated that a specific insertion in the *rpoB* gene confers RIF resistance, by the construction of a *Mtb* mutant harboring the same mutation.

Unfortunately, several *Mtb* clinical isolates resistant to new compounds used in TB therapy, such as bedaquiline (BDQ), have already spreaded worldwide (Bloemberg et al., 2015; Zimenkov et al., 2017). To better understand this phenomenon, Degiacomi et al. generated *Mtb* mutants resistant to BDQ starting from two MDR clinical isolates, miming what happens in clinical setting. These new BDQ resistant mutants harbored mutations in both *atpE* gene, coding for the target, and *Rv0678*, which encodes the repressor of MmpL5 efflux pump. The growth curves of BDQ resistant mutants were also evaluated. It was shown that *Rv0678* mutations could give an advantage in the growth rate, explaining the spread of BDQ resistance among *Mtb* clinical isolates also prior to BDQ treatment.

Further findings in the study of new TB drug targets and in the characterization of the existing ones are present in our collection.

In order to characterize GreA as new drug target, Feng et al. isolated *Mycobacterium smegmatis* and *Mtb* mutants and showed that knock-down *greA* mutant resulted in growth retardation in *Mtb* and it is essential for its survival under heat shock stress. Furthermore, these mutants are more susceptible to vancomycin and RIF. By RNA-seq, they showed the role of GreA in the metabolic regulation of mycobacteria.

Rohde and Sorci evaluated the possible synergy of NAD biosynthesis inhibitors with some antitubercular drugs for drug combination. In fact, several prodrugs require a NAD biosynthesis enzyme to be activated, such as INH, ethionamide, and delamanid.

This unique collection of articles in our Research Topic gives new insights into the characterization drug resistance mechanisms in *Mtb* as well as provides novel strategies to fight this notorious pathogen.

We would like to thank the reviewers for their comments that improved our manuscripts, and the authors for their excellent contributions.

Finally, we hope that this Collection would stimulate open discussions about this topic and new researches which will assist the scientific and pharmaceutical communities in finding promising weapons against TB.

## Author Contributions

All authors listed have made a substantial, direct and intellectual contribution to the work, and approved it for publication.

## Conflict of Interest

The authors declare that the research was conducted in the absence of any commercial or financial relationships that could be construed as a potential conflict of interest.
